# Causal relationship between intervertebral disc degeneration and neurological disorders: A Mendelian randomization study

**DOI:** 10.1097/MD.0000000000046797

**Published:** 2026-05-15

**Authors:** Xinhua Cao

**Affiliations:** a Dazhou Chinese Medicine Vocational College, Dazhou, Sichuan Province, China.

**Keywords:** intervertebral disc degeneration, Mendelian randomization, neurological disease, Parkinson disease, stroke

## Abstract

No study has assessed the association between intervertebral disc degeneration (IVDD) and neurological disorders. We explored their causal relationship using Mendelian randomization (MR). Genome-wide association study summary data on IVDD and 18 common neurological disorders from European populations were included. Univariate Mendelian randomization and multivariate Mendelian randomization were used to investigate the causal relationship between the 2. Three analyses were applied: inverse-variance-weighted (IVW), MR-Egger, and weighted median, with IVW as the primary analysis method. Sensitivity analyses included Cochran *Q*, MR-PRESSO, MR-Egger intercept, leave-one-out, and MR-Steiger tests. IVW methods showed that IVDD was associated with an increased risk of all-cause stroke (odds ratio [OR] = 1.127, 95% confidence interval [CI] = 1.027–1.236, *P* = .012) and ischemic stroke (OR = 1.127, 95% CI = 1.021–1.243, *P* = .018), IVDD was associated with a reduced risk of Parkinson disease (OR = 0.696, 95% CI = 0.537–0.903, *P* = .006), and the association of IVDD with the other 15 neurological disease categories was not significant. Sensitivity analyses supported this result. Further multivariate Mendelian randomization similarly confirmed the causal relationship of IVDD with all-cause stroke (OR = 1.123, 95% CI = 1.005–1.255, *P* = .041), ischemic stroke (OR = 1.120, 95% CI = 1.013–1.238, *P* = .026), Parkinson disease (OR = 0.668, 95% CI = 0.530–0.842, *P* = .001). IVDD is associated with increased stroke risk and reduced Parkinson disease risk. This study advances understanding and may inform clinical approaches, though further research is needed to elucidate underlying mechanisms.

## 1. Introduction

Neurological disorders encompass neurodegenerative diseases, cerebrovascular diseases, neuroinflammatory diseases, neuromuscular diseases, epilepsy, and other hereditary or acquired neurological disorders, which can lead to a wide range of symptoms, including motor deficits, sensory abnormalities, cognitive decline, and autonomic dysfunction.^[1–4]^ With population growth and aging, the burden of neurological disorders has increased dramatically, becoming the second leading cause of death globally as well as the leading cause of disability.^[5]^ To date, the causes and mechanisms of most neurological disorders are still unknown or not fully understood, and this limitation in understanding has led to the failure to achieve the desired therapeutic outcomes for most neurological disorders.^[6–8]^

Low back pain (LBP) is a common clinical condition that globally afflicts approximately 40% of the population during their lifetime and is currently the leading cause of the world’s disability burden.^[9,10]^ Repeated episodes of LBP can seriously affect patients’ quality of life as well as their mental health, and it has been estimated that the annual LBP-related expenditure in the United States exceeds $100 billion, creating an enormous economic and health burden on society.^[11]^ Intervertebral disc degeneration (IVDD) is a prevalent degenerative disease of the spine characterized by degeneration of the annulus fibrosus, dehydration of the nucleus pulposus, decreased levels of proteoglycans, and calcification of the cartilaginous endplates.^[12]^ IVDD is considered a major cause of LBP, and is also the pathological basis of many musculoskeletal disorders such as herniated discs, radiculopathies, spondylotic myelopathies, spinal stenosis, and vertebral body slippage.^[13,14]^

Previous studies have examined the association between LBP and certain neurological disorders: 2 cohort studies from Taiwan and Germany and a cross-sectional study from South Korea have shown a positive association between LBP and the occurrence of stroke, especially ischemic stroke;^[15–17]^ others have reported an association between LBP and several neurodegenerative disorders such as Alzheimer disease, Parkinson disease (PD), dementia, amyotrophic lateral sclerosis;^[18–22]^ in addition to this, one study demonstrated an association between LBP and neuroinflammatory diseases such as multiple sclerosis.^[23]^ However, there are also studies that show no association between LBP and several neurodegenerative diseases.^[24]^ In fact, there are many causes that can lead to LBP, like kidney stones or pyelonephritis, which can also present with LBP. It usually appears as a common symptom rather than a single disease, and most of the patients with LBP included in previous studies came from self-reporting, which means that there are many potential confounding factors that are difficult to control. We therefore decided to explore the association between IVDD, the main cause of LBP, and neurological disorders.

Mendelian randomization (MR) is an innovative epidemiological approach that uses genetic variants such as single nucleotide polymorphisms (SNPs) associated with an exposure of interest as instrumental variables (IVs) to explore possible causal associations between exposures and outcomes.^[25]^ The advantage of using the MR approach is that it allows causal inference in cases where randomized controlled trials are not feasible.^[26]^ In addition to this, the fact that genetic variants are randomly assigned at conception and remain with us throughout our lives means that they can minimize the influence of confounding factors such as behavioral, social, and environmental factors, as well as avoiding reverse causality between risk factors and disease.^[27]^ Genome-wide association study (GWAS) is a genetic research method that enables the identification of IVs associated with specific exposures. The ability to identify IVs and the use of rigorous methods to avoid the effects of population stratification and confounders greatly facilitate MR studies, allowing us to use published GWAS results for MR analyses.^[28]^

Our aim was to identify genetic associations between IVDD and 18 common neurological disorders by MR analysis.

## 2. Materials and methods

### 2.1. Study design

**Figure 1.**
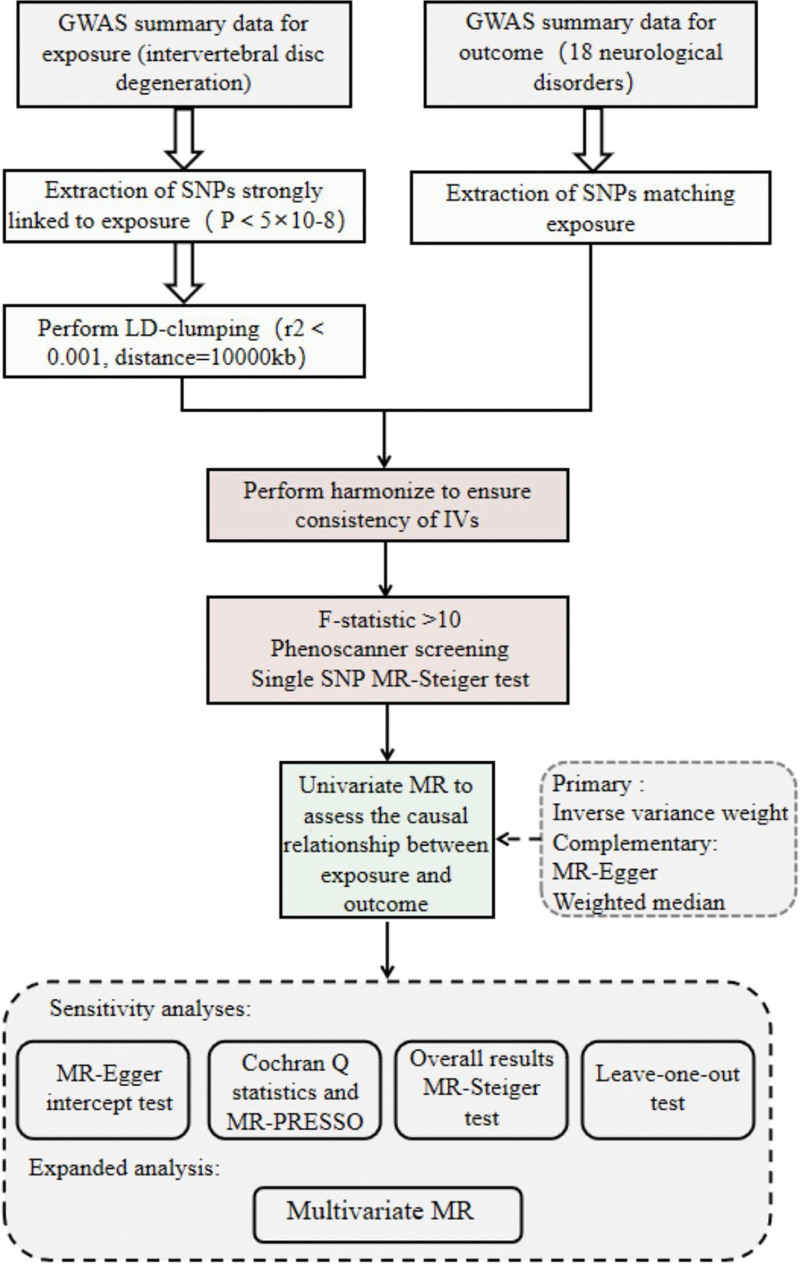
Flowchart of MR analysis of the causal effect of IVDD on neurological disease. IVDD = intervertebral disc degeneration.

In this study, we used a two-sample MR analysis approach, first using univariate Mendelian randomization (UVMR) to determine whether there is a causal relationship between IVDD and psychiatric system disorders, and then using multivariate Mendelian randomization (MVMR) to further verify the reliability of their relationship. The reliability of the results of the MR analyses depended on the use of rigorous criteria for the selection of the IVs. First, the IVs had to be selected in a way that satisfied 3 key assumptions: there is a strong association between the IVs and our exposure factor of interest; the IVs cannot be associated with any other potential confounders that may affect exposure and outcome; the IVs affect outcomes only through IVDD. Additionally, the effects of population stratification and sample overlap had to be fully avoided in the selection of IVs, so we focused only on European ancestry, and the GWAS data on exposure and outcome were from different consortium cohorts. The reporting of this study followed the Strengthening the Reporting of Observational Studies in Epidemiology Using Mendelian Randomization guidelines.^[29]^ Ethical approval was obtained for all previously published GWAS summary data we used; hence, no additional ethical approval was required. Figure 1 shows an overview of the study design.

### 2.2. Data sources

#### 2.2.1. IVs selection

GWAS summary data for the IVDD were obtained from the FinnGen consortium (https://www.finngen.fi/fi), GWAS ID: finn-b-M13_INTERVERTEB, which contains 20,001 cases and 164,682 controls of individuals of European ancestry. The IVDD was measured using radiological imaging (e.g., MRI and CT scans), standardized clinical assessments, patient-reported outcomes for measurement, and diagnostic criteria based on the International Classification of Diseases ICD-10 code M51 (defined as: thoracic disc, thoracolumbar disc, and lumbosacral disc disease), ICD-9 code 722 (defined as: intervertebral disc disease), and ICD-8 code 275 (defined as: intervertebral disc displacement).^[30]^ To satisfy the 3 key assumptions for the selection of IVs for MR, we established the following criteria that were rigorously applied to screen SNPs as IVs for this study: SNPs with *P* < 5 × 10^‐8^ were selected, which are considered to be SNPs strongly correlated with our exposure factor of interest.^[25]^ To avoid SNPs from being affected by linkage disequilibrium (LD) among them, the European population samples were consulted to LD-clumping was performed to exclude SNPs with strong LD,^[31]^ and widely used thresholds such as *r*^2^ < 0.001 and distance = 10,000 kb were used,^[32]^ usually variables with *F*-statistic > 10 were defined as strong IVs,^[33]^ mand the formula *F*-statistic = beta2/se2 to calculate the strength of each selected SNP, and SNPs with *F*-statistic < 10 will be excluded; to avoid the influence of potential confounders, the screened SNPs were manually checked in Phenoscanner (www.phenoscanner.medschl.cam.ac.uk), and SNPs associated with potential confounders (e.g., smoking, body mass index, hypertension, diabetes, lipid levels, and inflammatory markers) were excluded;^[34]^ (5palindromic SNPs were excluded to avoid bias caused by strand ambiguity.

#### 2.2.2. Neurological diseases

The GWAS summary data for the 18 common neurological disorders included in this study were derived from European ancestries. GWAS summary data for PD were obtained from the International Parkinson’s Disease Genomics Consortium.^[35]^ The International Multiple Sclerosis Genetics Consortium provided GWAS summary data on multiple sclerosis.^[36]^ GWAS summary data on meningitis were obtained from the MRC Integrative Epidemiology Unit.^[28]^ The GWAS summary data on osteomyelitis were provided by UK Biobank.^[37]^ Osteomyelitis was included because it can involve the central nervous system as a complication (e.g., spinal epidural abscess). GWAS summary data for cerebral hemorrhage, subarachnoid hemorrhage, epilepsy, and Bell palsy were all obtained from the same GWAS study.^[38]^ A GWAS meta-analysis for stroke provided GWAS summary data for all-cause stroke and ischemic stroke.^[39]^ GWAS summary data for lacunar stroke,^[40]^ Alzheimer disease,^[41]^ amyotrophic lateral sclerosis,^[42]^ dementia with Lewy bodies,^[43]^ frontotemporal lobe dementia,^[44]^ myasthenia gravis,^[45]^ migraine headaches,^[46]^ and neuroblastoma^[47]^ were obtained from their respective relevant GWAS studies. Table 1 shows details of the GWAS summary data for the neurological disorders included in this study.

**Table 1:** Detailed information on GWAS summary data for neurological disorders.

Trait	GWAS ID	Sample size	ncase	Number of SNPs	Consortium/PMID
Stroke	ebi-a-GCST005838	446,696	40,585	446,696	29531354
Ischemic stroke	ebi-a-GCST005843	440,328	34,217	7,537,579	29531354
Lacunar stroke	ebi-a-GCST90014122	248,929	6030	6,909,434	33773637
Intracerebral hemorrhage	ebi-a-GCST90018870	473,513	1935	24,191,284	34594039
Subarachnoid hemorrhage	ebi-a-GCST90018923	473,255	1693	24,191,735	34594039
Alzheimer disease	ebi-a-GCST90027158	487,511	39,106	20,921,626	35379992
Parkinson disease	ieu-b-7	482,730	33,674	17,891,936	International Parkinson’s Disease Genomics Consortium
Amyotrophic lateral sclerosis	ebi-a-GCST90027163	138,086	27,205	10,426,600	34873335
Dementia with Lewy bodies	ebi-a-GCST90001390	6618	2591	7593,175	33589841
Frontotemporal dementia	ieu-b-43	3024	515	494,577	20154673
Myasthenia gravis	ebi-a-GCST90093061	38,243	1873	23,679,120	35074870
Multiple sclerosis	ieu-b-18	115,803	47,429	6,304,359	International Multiple Sclerosis Genetics Consortium
Meningitis	ukb-b-10224	462,933	1909	9,851,867	MRC-IEU
Migraine	ebi-a-GCST90038646	484,598	13,971	9,587,836	33959723
Epilepsy	ebi-a-GCST90018840	458,310	4382	24,186,492	34594039
Neuroblastoma	ieu-a-816	4881	1627	468,788	23222812
Bell palsy	ebi-a-GCST90018843	463,240	1894	24,193,930	34594039
Osteomyelitis	ieu-b-4975	486,484	4836	12,243,512	UK Biobank

GWAS = genome-wide association, MRC-IEU = MRC Integrative Epidemiology Unit, SNP = single-nucleotide polymorphism.

### 2.3. Statistical analysis

Three analytical methods were used in our study to assess the causal relationship that exists between IVDD and 18 neurological disorders: inverse variance weight (IVW), MR-Egger, and weighted median (WM). The possibility that IVs may directly affect the outcome rather than indirectly through exposure factors is referred to as horizontal pleiotropy. The IVW, MR-Egger, and WM methods make different assumptions about the possible horizontal pleiotropy of IVs. The IVW method assumes that there is no horizontal pleiotropy for all IVs, and it averages the estimated effect values of the individual IVs according to the weighted average of their inverse variances to obtain an estimate of the causal effect of the IVs as a whole on the outcome;^[48]^ in contrast, the MR-Egger method assumes horizontal pleiotropy for all IVs, and causal effects can be estimated even in the presence of bias.^[49]^ WM assumes horizontal polytropy for 50% of the IVs, which provides reliable estimates even in the presence of level bias for a few IVs.^[50]^ In summary, in the absence of horizontal pleiotropy, the causality assessed by the IVW method is the most reliable, and therefore its results were used by us as the primary outcome. The results of both MR-Egger and WM methods were referred to if the effect of horizontal pleiotropy was present.

To assess horizontal pleiotropy while avoiding heterogeneity, excessive influence of individual IVs on the overall results, and reverse causality, we conducted a series of sensitivity analyses, all of which aimed to ensure the robustness of the results of the MR analyses. Using MR-Egger regression, we assessed whether IVs were horizontally pleiotropic based on MR-Egger intercept.^[49]^ Significant differences or variations in the effects of IVs on outcomes are referred to as heterogeneity, which may arise from differences in biological effects between IVs.Cochran *Q* statistics were used to identify whether there was heterogeneity between IVs, and if there was heterogeneity, IVs that may be heterogeneous were analyzed using MR-PRESSO, and, after manual exclusion, re-MR analysis was.^[51,52]^ Leave-one-out (LOO) analyses are able to assess the robustness of causal effects and the unique contribution of each IV by excluding IVs one by one, helping to improve the accuracy and reliability of causal inferences. The MR-Steiger test is used to test whether IVs are likely to be affected by the outcome, thus avoiding interference from reverse causation. The effect between each IV and genotype can also be assessed so as to assess which direction is more plausible and credible; if the MR-Steiger test shows that the IV has a significant effect on the genotype, while the genotype has a nonsignificant effect on the IV, this will enhance the credibility of the selected IVs as causal variables.^[53]^

In order to eliminate some of the possible biases and thus draw more reliable conclusions, we further validated the causal relationships derived from UVMR using MVMR, an extension of MR analysis for assessing the effects of multiple exposures on the outcome variable at the same time, where we can obtain the direct effect of each exposure factor in the model on the outcome variable that is not confounded or mediated by the other exposures.^[54]^

All our analyses were performed in R (4.4.1) software using the 3 R packages TwoSampleMR, MR-PRESSOR, and MendelianRandomization.

## 3. Results

### 3.1. Instrumental variable

After adhering to the previously established strict criteria for screening SNPs associated with IVDD, 6 SNPs were included in our study as IVs. All the selected SNPs were strong IVs with an *F*-statistic >10 and high statistical power. No palindromic SNPs with symmetry were found when MR analysis was performed. Detailed information on the SNPs included in the study can be seen in Table S1.

### 3.2. Univariate Mendelian randomization

**Figure 2.**
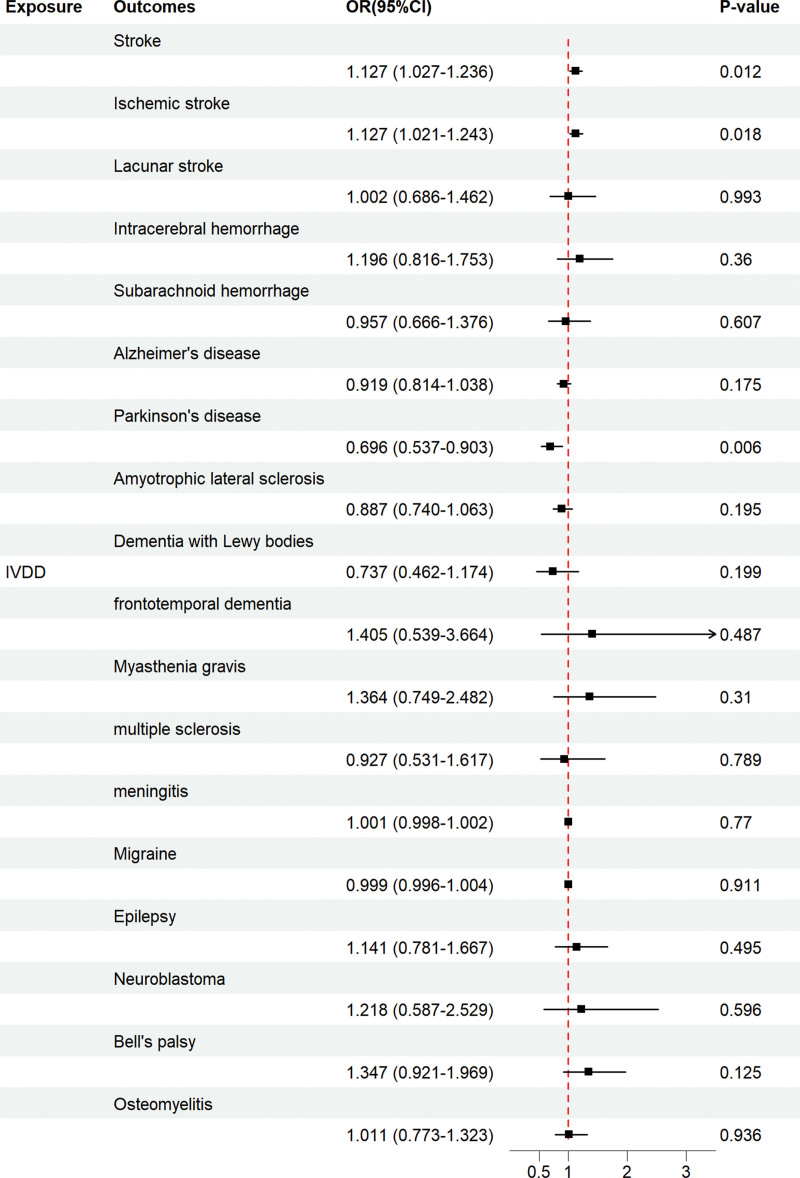
The forest plot depicts the findings of the UVMR on the causal effects of IVDD on neurological disease. CI = confidence interval; IVDD = intervertebral disc degeneration; OR = odds ratio; *P*-value = *P* value of the causal estimate; UVMR = univariate Mendelian randomization.

IVW methods showed that IVDD was associated with an increased risk of all-cause stroke (odds ratio [OR] = 1.127, 95% confidence interval [CI] = 1.027–1.236, *P* = .012) and ischemic stroke (OR = 1.127, 95% CI = 1.021–1.243, *P* = .018), IVDD was associated with a reduced risk of PD (OR = 0.696, 95% CI = 0.537–0.903, *P* = .006), and the association of IVDD with the other 15 categories of neurological disorders was not significant. The results of subsequent sensitivity analyses of IVDD with all-cause stroke, ischemic stroke, and PD also supported the causal relationship between the genetic prediction of the IVW method: the *P*-values in the Cochran *Q* statistics were all >.05, suggesting that there was no heterogeneity, and the *P*-values in the MR-Egger intercept test were >.05, suggesting that there was no horizontal pleiotropy. indicating the absence of horizontal pleiotropy, and therefore we consider the results from the IVW method to be the most reliable; the results of the LOO test all indicate that the overall findings are not driven by a single SNP; and the results of the MR-Steiger test analyses validate the correctness of the direction of causality in the 3 groups, as well as the direction of the selected IVs with respect to the genotypes. Figure 2 shows the results of IVW analyses in this study. The results of WM analyses and MR-Egger analyses are listed in Table S2. The specific results of the analyses of Cochran *Q* statistics, the MR-Egger intercept, and the MR-Steiger test can be seen in Tables S2 to S5 respectively. Figures S1 to S3, show the scatterplot, funnel plot, and LOO plot of the UVMR results in this study, respectively.

### 3.3. Multivariate Mendelian randomization

**Figure 3.**
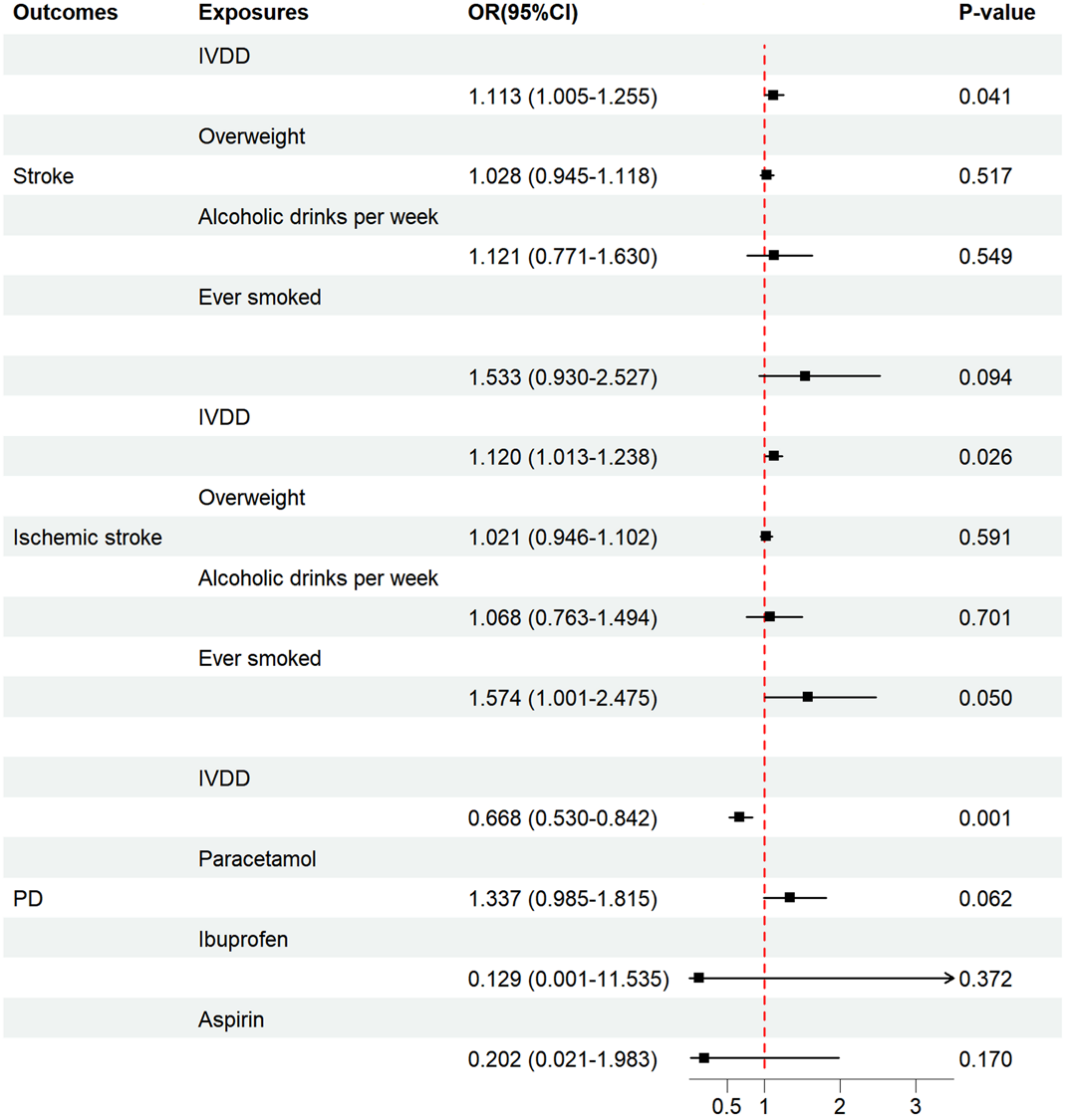
The forest plot depicts the findings of the MVMR on the causal effects of IVDD on all-cause stroke, ischemic stroke, and Parkinson disease. CI = confidence interval; IVDD = intervertebral disc degeneration; MVMR = multivariate Mendelian randomization; OR = odds ratio; *P*-value = *P* value of the causal estimate.

Smoking, alcohol consumption, and overweight are 3 common shared risk factors for IVDD and stroke.^[55,56]^ After we coordinated smoking, alcohol consumption, and overweight in the model of IVDD with stroke and ischemic stroke, the IVW method showed that a positive causal relationship between IVDD and the risk of all-cause stroke (OR = 1.123, 95% CI = 1.005–1.255, *P* = .041) and ischemic stroke risk (OR = 1.120, 95% CI = 1.013–1.238, *P* = .026) remained positively and causally associated with each other. In the MVMR model of IVDD and PD, we mainly considered the possible influence of nonsteroidal anti-inflammatory drugs (NSAIDs) on the causal relationship between the 2; NSAIDs are commonly used for IVDD-related conditions such as low back pain, and previous studies have suggested that NSAID use may be a protective factor for PD.^[57]^ After harmonizing aspirin, ibuprofen, and paracetamol, the IVW method showed that there was still a negative causal association between IVDD and PD risk (OR = 0.668, 95% CI = 0.530–0.842, *P* = .001). Figure 3 illustrates the results of the MVMR analysis. Detailed GWAS summary data for covariates can be seen in Table S6.

## 4. Discussion

A number of previous studies have reported the association that exists between LBP and a variety of neurologic disorders, whereas IVDD, which is the most prominent cause of LBP, has not yet been explored in studies exploring its relationship with neurologic disorders. We evaluated the possible causal relationship between IVDD and 18 common neurological diseases by MR analysis. The findings suggest that IVDD is associated with an increased risk of all-cause stroke, ischemic stroke, and also with a reduced risk of PD. However, the association with lacunar stroke, cerebral hemorrhage, subarachnoid hemorrhage, Alzheimer disease, amyotrophic lateral sclerosis, dementia with Lewy bodies, frontotemporal dementia, myasthenia gravis, multiple sclerosis, meningitis, migraine, epilepsy, neuroblastoma, Bell palsy, and osteomyelitis was not significant. In a subsequent MVMR model that adjusted for smoking, alcohol consumption, overweight, and several covariates (aspirin, ibuprofen, and paracetamol), the genetically predicted causal relationships between IVDD and all-cause stroke, ischemic stroke, and PD remained statistically significant. These findings provide new perspectives for understanding the underlying biological mechanisms between IVDD and neurologic disorders.

Several previous studies have found LBP to be associated with stroke risk, and a Taiwanese cohort study that included 10,308 patients with LBP and a matched group of 20,616 non-LBP patients with a mean follow-up time of 8 years showed that patients with LBP had an increased risk of stroke, especially ischemic stroke;^[15]^ a German cohort study including 159,440 patients with 10 years of follow-up showed that LBP was positively associated with stroke risk,^[16]^ but was significantly related only to all-cause stroke and ischemic stroke and not to hemorrhagic stroke; in a Korean cross-sectional study including 17,038 participants, researchers found that stroke was positively associated with LBP.^[17]^ Our MR analysis found for the first time that IVDD, the main cause of LBP, had a positive causal association with all-cause stroke and ischemic stroke, but not with lacunar stroke, cerebral hemorrhage, and subarachnoid hemorrhage, among several other types of stroke. The specific association with all-cause and ischemic stroke, but not hemorrhagic stroke, may be due to different underlying pathophysiological mechanisms: ischemic stroke is often related to atherosclerosis, thrombosis, and embolism, while hemorrhagic stroke involves vessel rupture. The factors associated with the increased risk of stroke in IVDD, especially ischemic stroke, are poorly known, although they may involve spinal cord injury (SCI), pain stimulation, activity limitations, spinal surgery complications, and an inflammatory response.

IVDD results in osteophytes, herniated discs, and slipped vertebrae, all of which may cause compression of the adjacent spinal cord or nerve roots, leading to SCI.^[58]^ A cohort study reported that patients with SCI were more than twice as likely to have a stroke compared to the general population, and the type of stroke was further categorized into ischemic and hemorrhagic in the SCI group, with ischemic strokes being 3.42 times more common than hemorrhagic strokes.^[59]^ In addition, the predominant symptoms for patients with IVDD are chronic pain and limitation of spinal mobility,^[60]^ and several previous studies have shown that chronic pain is associated with an increased risk of stroke.^[61,62]^ Whereas physical activity limitation, whether due to chronic pain or limited spinal mobility, may be a possibility for IVDD leading to an increased risk of stroke, physical activity is a modifiable risk factor included in the guidelines for primary prevention of stroke,^[63]^ and a large number of studies have demonstrated that an increase in physical activity reduces the risk of stroke even with more moderate physical activities such as leisure activities and work commuting.^[64,65]^ In addition, the number of patients with IVDD undergoing surgery has increased progressively with aging, and stroke complications after spinal surgery may be partly responsible for this association, although they are relatively rare.^[66–68]^ Finally, degenerated intervertebral discs spontaneously produce a number of inflammatory factors such as interleukin-1, interleukin-6, tumor necrosis factor-α, prostaglandin E2, etc, and these inflammatory factors also play an important role in the development of stroke.^[69,70]^

PD is a chronic progressive movement disorder characterized by neuronal degeneration in the central nervous system, and several studies have shown that musculoskeletal pain such as LBP is frequently observed in patients with PD, in addition to which scoliosis is more common in patients with PD than in the general population, and the cause of this may be related to biomechanical and neuromuscular factors such as dystonia.^[71,72]^ There was also a study evaluating the association of PD with LBP that showed that approximately 40% of patients developed LBP before the diagnosis of PD.^[20]^ There was also a prospective study evaluating LBP and the risk of dementia, which suggested that LBP may be helpful in maintaining cognitive function.^[21]^ However, these are observational studies with small sample sizes that do not provide direct causal evidence. Whereas LBP has many etiologic factors, it usually appears as a common symptom rather than a single disease, and patients’ LBP is also mostly self-reported, which means that there are many potential confounders that are difficult to control for, which may explain the conflicting conclusions in previous studies. Interestingly, our MR study confirmed that IVDD is associated with a reduced risk of PD.

Our study considered the effect of some common drug use, such as NSAIDs, on this association. In our MVMR, we adjusted for aspirin and ibuprofen (NSAIDs) and paracetamol (a non-NSAID analgesic). The inclusion of paracetamol was based on its common use for pain relief in IVDD, though it is not an NSAID. The results showed that these adjustments did not significantly alter the association between IVDD and PD, so we do not believe that NSAID use mediated this association. In addition, there may be some other common therapies between IVDD and PD, such as acupuncture and spinal cord stimulation, which have been widely used in the treatment of chronic pain, including LBP and back pain.^[73,74]^ Although there are many studies reporting the effectiveness of these treatment modalities in treating PD, there is generally a low level of evidence and no studies confirming that these treatments can reduce PD.^[75,76]^ One possible mechanism for the causal relationship between the 2 is that chronic pain stimuli, like discogenic or radicular pain, lead to central sensitization and induce the release of endogenous neuroprotective substances such as brain-derived neurotrophic factor, nerve growth factor, which have long been shown to be beneficial for PD.^[77–79]^ In addition, whether some pathological changes caused by IVDD, such as nerve root compression or spinal cord compression, could somehow produce stimulation of brain regions closely related to PD, such as the substantia nigra and the basal nuclei, thereby slowing down the degeneration and death of substantia nigra dopaminergic neurons, is one of the speculations about the association between IVDD and reduced risk of PD. We acknowledge that reverse causality (i.e., PD leading to IVDD) is a potential concern, but our MR-Steiger test and other sensitivity analyses did not support reverse causality. Future studies are needed to explore the precise mechanisms.

Globally, stroke and PD have become a major public health challenge. Stroke is one of the leading causes of death and disability, with ischemic stroke accounting for 75% to 80% of stroke cases.^[80]^ Despite the extensive research on stroke over the past decades and the reduction of stroke mortality in some regions, there is still a lack of effective and simple treatment and prevention options. Therefore, exploring new therapies as well as continuing to clarify the pathogenesis of stroke remains a major current research direction.^[81]^ Similarly, the incidence of PD, a complex neurodegenerative disease, is increasing every year, and the number of Parkinson disease cases is predicted to double in the next 3 decades.^[82]^ Alarmingly, no therapeutic options have been found that can effectively stop or slow the progression of the disease.^[83]^ This state of affairs suggests an urgent need for an in-depth study of its etiology and pathogenesis, and only through the resolution of these fundamental issues can we lay a sound foundation for future prevention and treatment strategies.

In conclusion, our findings identify for the first time that IVDD is a risk factor for all-cause stroke and ischemic stroke and a protective factor for PD. This finding may contribute to our understanding of the complex relationship between these diseases and spark future interest in exploring the underlying biological mechanisms behind these associations, leading to new ideas for the prevention and treatment of these diseases. We suggest that clinicians should consider intervertebral disc-related lesions in addition to cardiovascular and cerebrovascular risk factors when assessing stroke risk.

Our study has several strengths. First, we investigated for the first time the causal relationship between IVDD and 18 common neurological disorders and confirmed the causal relationship between IVDD and all-cause stroke, ischemic stroke, and PD. Second, the use of a novel epidemiologic method such as MR can largely avoid the influence of confounding factors and is also highly economical. In addition, the robustness of our results is greatly enhanced by the strict adherence to MR reporting guidelines, the selection of the most recent and largest sample size, the most rigorous screening of IVs, and a series of sensitivity tests, including the use of MVMR to exclude potential confounders that are not easily observable.

At the same time, there are some shortcomings of this study that must be acknowledged. First, due to data limitations, we were unable to analyze samples of ancestry other than European ancestry, which limits the generalization of our findings to other populations. Secondly, some of the outcome data included in the study, such as frontotemporal dementia, had a small sample size, which may have led to some bias in the results of that part of the study. In addition, although we considered confounders and horizontal pleiotropy, none of the MR studies could ensure that these biases were fully excluded. Finally, the diagnostic criteria for GWAS data for IVDD were based on ICD-10 rather than ICD-11 and thus may have some shortcomings.

## 5. Conclusions

In conclusion, our study highlights that IVDD is associated with an increased risk of all-cause stroke and ischemic stroke and a decreased risk of PD. This study may play a positive role in the development of this field and provide new ideas and approaches for clinical treatment, and further studies are needed in the future to reveal the potential mechanisms of action between the 2.

## Acknowledgments

We sincerely thank the consortium that provided the GWAS data.

## Author contributions

**Conceptualization:** Xinhua Cao.

**Funding acquisition:** Xinhua Cao.

**Methodology:** Xinhua Cao.

**Software:** Xinhua Cao.

**Supervision:** Xinhua Cao.

**Writing – original draft:** Xinhua Cao.

**Writing – review & editing:** Xinhua Cao.







**Figure:**
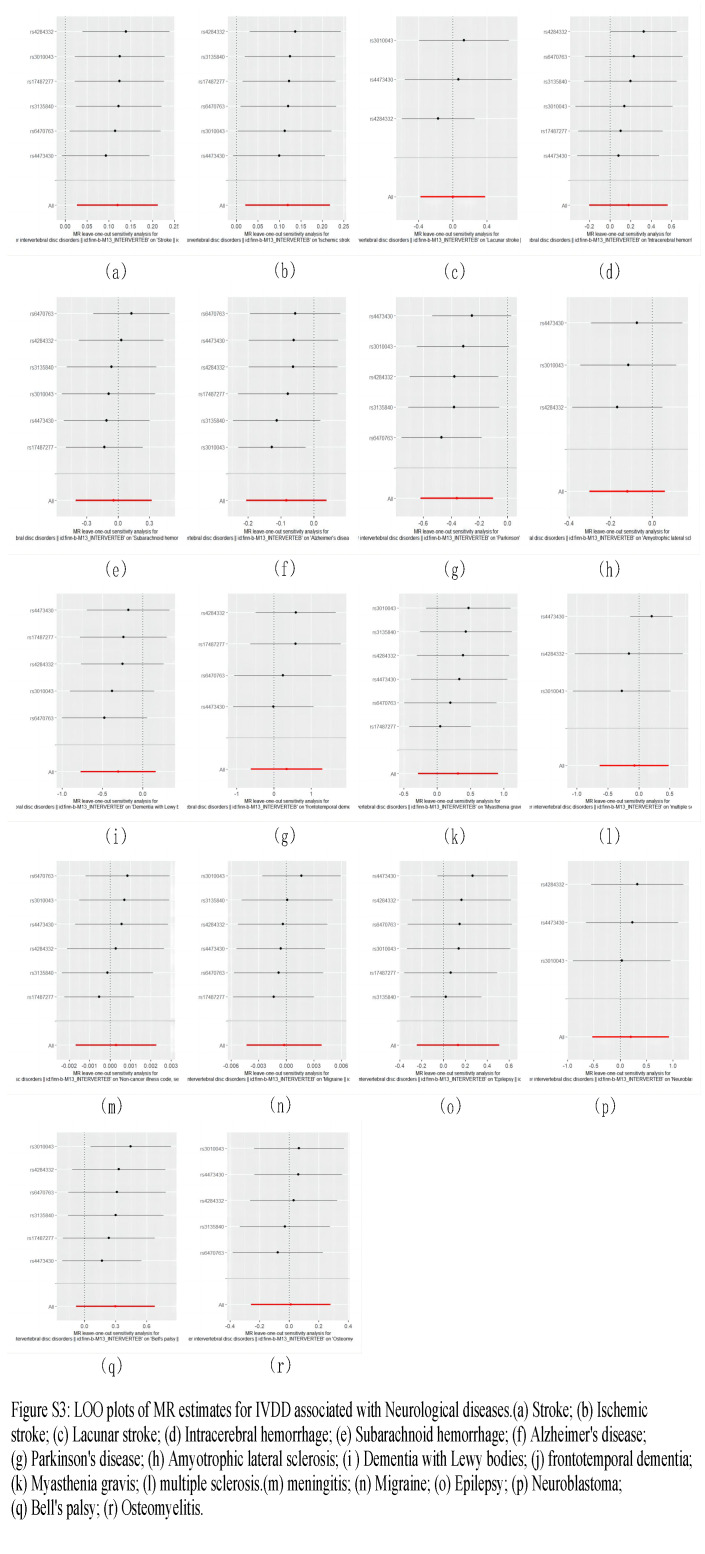


## References

[1] Armstrong MJ Okun MS. Diagnosis and treatment of Parkinson disease a review. JAMA. 2020;323:548–60. 32044947 10.1001/jama.2019.22360

[2] Campbell BCV De Silva DA Macleod MR. Ischaemic stroke. Nat Rev Dis Primers. 2019;5:70. 31601801 10.1038/s41572-019-0118-8

[3] Dobson R Giovannoni G. Multiple sclerosis – a review. Eur J Neurol. 2019;26:27–40. 30300457 10.1111/ene.13819

[4] Gilhus NE Tzartos S Evoli A Palace J Burns TM Verschuuren JJGM. Myasthenia gravis. Nat Rev Dis Primers. 2019;5:30. 31048702 10.1038/s41572-019-0079-y

[5] GBD 2016 Neurology Collaborators. Global, regional, and national burden of neurological disorders, 1990–2016: a systematic analysis for the Global Burden of Disease Study 2016. Lancet Neurol. 2019;18:459–80. 30879893 10.1016/S1474-4422(18)30499-X PMC6459001

[6] Jankovic J Tan EK. Parkinson’s disease: etiopathogenesis and treatment. J Neurol Neurosurg Psychiatry. 2020;91:795–808. 32576618 10.1136/jnnp-2019-322338

[7] Passeri E Elkhoury K Morsink M. Alzheimer’s disease: treatment strategies and their limitations. Int J Mol Sci. 2022;23:13954. 36430432 10.3390/ijms232213954 PMC9697769

[8] Kiernan MC Vucic S Cheah BC. Amyotrophic lateral sclerosis. Lancet. 2011;377:942–55. 21296405 10.1016/S0140-6736(10)61156-7

[9] Buchbinder R van Tulder M Öberg B. Low back pain: a call for action. Lancet. 2018;391:2384–8. 29573871 10.1016/S0140-6736(18)30488-4

[10] Foster NE Anema JR Cherkin D. Prevention and treatment of low back pain: evidence, challenges, and promising directions. Lancet. 2018;391:2368–83. 29573872 10.1016/S0140-6736(18)30489-6

[11] Katz JN. Lumbar disc disorders and low-back pain: socioeconomic factors and consequences. J Bone Joint Surg Am. 2006;88:21–4. 16595438 10.2106/JBJS.E.01273

[12] Vergroesen PP Kingma I Emanuel KS. Mechanics and biology in intervertebral disc degeneration: a vicious circle. Osteoarthritis Cartilage. 2015;23:1057–70. 25827971 10.1016/j.joca.2015.03.028

[13] GBD 2019 Diseases and Injuries Collaborators. Global burden of 369 diseases and injuries in 204 countries and territories, 1990–2019: a systematic analysis for the Global Burden of Disease Study 2019. Lancet. 2020;396:1204–22. 33069326 10.1016/S0140-6736(20)30925-9 PMC7567026

[14] Wang Y Che M Xin J Zheng Z Li J Zhang S. The role of IL-1β and TNF-α in intervertebral disc degeneration. Biomed Pharmacother. 2020;131:110660. 32853910 10.1016/j.biopha.2020.110660

[15] Wang HC Su Y-C Luk H-N Wang J-H Hsu C-Y Lin S-Z. Increased risk of strokes in patients with chronic low back pain (CLBP): A nationwide population-based cohort study. Clin Neurol Neurosurg. 2020;192:105725. 32086183 10.1016/j.clineuro.2020.105725

[16] Jacob L Smith L Koyanagi A. Chronic low back pain and incident transient ischemic attack and stroke in general practices in Germany. Healthcare (Basel). 2023;11:1499. 37239785 10.3390/healthcare11101499 PMC10217849

[17] Park HJ Choi JY Lee WM Park SM. Prevalence of chronic low back pain and its associated factors in the general population of South Korea: a cross-sectional study using the National Health and Nutrition Examination Surveys. J Orthop Surg Res. 2023;18:29. 36631903 10.1186/s13018-023-03509-x PMC9832776

[18] Udeh-Momoh C Price G Ropacki MT. Prospective evaluation of cognitive health and related factors in elderly at risk for developing alzheimer’s dementia: a longitudinal cohort study. J Prev Alzheimers Dis. 2019;6:256–66. 31686098 10.14283/jpad.2019.31

[19] Ozturk EA Kocer BG. Predictive risk factors for chronic low back pain in Parkinson’s disease. Clin Neurol Neurosurg. 2018;164:190–5. 29272805 10.1016/j.clineuro.2017.12.011

[20] Silveira Barezani AL de Figueiredo Feital AMB Gonçalves BM Christo PP Scalzo PL. Low back pain in Parkinson’s disease: a cross-sectional study of its prevalence, and implications on functional capacity and quality of life. Clin Neurol Neurosurg. 2020;194:105787. 32244035 10.1016/j.clineuro.2020.105787

[21] Yamada K Kubota Y Tabuchi T. A prospective study of knee pain, low back pain, and risk of dementia: the JAGES project. Sci Rep. 2019;9:10690. 31337809 10.1038/s41598-019-47005-x PMC6650603

[22] Aggarwal SP Zinman L Simpson E. Safety and efficacy of lithium in combination with riluzole for treatment of amyotrophic lateral sclerosis: a randomised, double-blind, placebo-controlled trial. Lancet Neurol. 2010;9:481–8. 20363190 10.1016/S1474-4422(10)70068-5 PMC3071495

[23] Massot C Donze C Guyot MA Leteneur S. Low back pain in patients with multiple sclerosis: a systematic review and the prevalence in a French multiple sclerosis population. Rev Neurol (Paris). 2021;177:349–58. 33032798 10.1016/j.neurol.2020.07.018

[24] Wu PF Du B Wang B. Joint analysis of genome-wide association data reveals no genetic correlations between low back pain and neurodegenerative diseases. Front Genet. 2021;12:744299. 34630533 10.3389/fgene.2021.744299 PMC8493037

[25] Davies NM Holmes MV Davey Smith G. Reading Mendelian randomisation studies: a guide, glossary, and checklist for clinicians. BMJ. 2018;362:k601. 30002074 10.1136/bmj.k601 PMC6041728

[26] Sekula P Del Greco MF Pattaro C Köttgen A. Mendelian randomization as an approach to assess causality using observational data. J Am Soc Nephrol. 2016;27:3253–65. 27486138 10.1681/ASN.2016010098 PMC5084898

[27] Richmond RC Davey Smith G. Mendelian randomization: concepts and scope. Cold Spring Harb Perspect Med. 2022;12:a040501. 34426474 10.1101/cshperspect.a040501 PMC8725623

[28] Hemani G Zheng J Elsworth B. The MR-Base platform supports systematic causal inference across the human phenome. Elife. 2018;7:e34408. 29846171 10.7554/eLife.34408 PMC5976434

[29] Skrivankova VW Richmond RC Woolf BAR. Strengthening the reporting of observational studies in epidemiology using mendelian randomisation (STROBE-MR): explanation and elaboration. BMJ. 2021;375:n2233. 34702754 10.1136/bmj.n2233 PMC8546498

[30] Kurki MI Karjalainen J Palta P. FinnGen provides genetic insights from a well-phenotyped isolated population. Nature. 2023;613:508–18. 36653562 10.1038/s41586-022-05473-8 PMC9849126

[31] Pistis G Porcu E Vrieze SI. Rare variant genotype imputation with thousands of study-specific whole-genome sequences: implications for cost-effective study designs. Eur J Hum Genet. 2015;23:975–83. 25293720 10.1038/ejhg.2014.216 PMC4463504

[32] Burgess S Davey Smith G Davies NM. Guidelines for performing Mendelian randomization investigations: update for summer 2023. Wellcome Open Res. 2019;4:186. 32760811 10.12688/wellcomeopenres.15555.1 PMC7384151

[33] Burgess S Thompson SG; CRP CHD Genetics Collaboration. Avoiding bias from weak instruments in Mendelian randomization studies. Int J Epidemiol. 2011;40:755–64. 21414999 10.1093/ije/dyr036

[34] Kamat MA Blackshaw JA Young R. PhenoScanner V2: an expanded tool for searching human genotype–phenotype associations. Bioinformatics. 2019;35:4851–3. 31233103 10.1093/bioinformatics/btz469 PMC6853652

[35] Nalls MA Blauwendraat C Vallerga CL. Identification of novel risk loci, causal insights, and heritable risk for Parkinson’s disease: a meta-analysis of genome-wide association studies. Lancet Neurol. 2019;18:1091–102. 31701892 10.1016/S1474-4422(19)30320-5 PMC8422160

[36] International Multiple Sclerosis Genetics Consortium. Multiple sclerosis genomic map implicates peripheral immune cells and microglia in susceptibility. Science. 2019;365:1417. 10.1126/science.aav7188 PMC7241648 31604244

[37] Sudlow C Gallacher J Allen N. UK biobank: an open access resource for identifying the causes of a wide range of complex diseases of middle and old age. PLoS Med. 2015;12:e1001779. 25826379 10.1371/journal.pmed.1001779 PMC4380465

[38] Sakaue S Kanai M Tanigawa Y. A cross-population atlas of genetic associations for 220 human phenotypes. Nat Genet. 2021;53:1415–24. 34594039 10.1038/s41588-021-00931-x PMC12208603

[39] Malik R Chauhan G Traylor M. Multiancestry genome-wide association study of 520,000 subjects identifies 32 loci associated with stroke and stroke subtypes. Nat Genet. 2018;50:524–37. 29531354 10.1038/s41588-018-0058-3 PMC5968830

[40] Traylor M Persyn E Tomppo L. Genetic basis of lacunar stroke: a pooled analysis of individual patient data and genome-wide association studies. Lancet Neurol. 2021;20:351–61. 33773637 10.1016/S1474-4422(21)00031-4 PMC8062914

[41] Bellenguez C Küçükali F Jansen IE. New insights into the genetic etiology of Alzheimer’s disease and related dementias. Nat Genet. 2022;54:412–36. 35379992 10.1038/s41588-022-01024-z PMC9005347

[42] van Rheenen W van der Spek RAA Bakker MK. Common and rare variant association analyses in amyotrophic lateral sclerosis identify 15 risk loci with distinct genetic architectures and neuron-specific biology. Nat Genet. 2021;53:1636–48. 34873335 10.1038/s41588-021-00973-1 PMC8648564

[43] Chia R Sabir MS Bandres-Ciga S. Genome sequencing analysis identifies new loci associated with Lewy body dementia and provides insights into its genetic architecture. Nat Genet. 2021;53:294–303. 33589841 10.1038/s41588-021-00785-3 PMC7946812

[44] Van Deerlin VM Sleiman PMA Martinez-Lage M. Common variants at 7p21 are associated with frontotemporal lobar degeneration with TDP-43 inclusions. Nat Genet. 2010;42:234–9. 20154673 10.1038/ng.536 PMC2828525

[45] Chia R Saez-Atienzar S Murphy N. Identification of genetic risk loci and prioritization of genes and pathways for myasthenia gravis: a genome-wide association study. Proc Natl Acad Sci U S A. 2022;119:e2108672119. 35074870 10.1073/pnas.2108672119 PMC8812681

[46] Dönertaş HM Fabian DK Valenzuela MF Partridge L Thornton JM. Common genetic associations between age-related diseases. Nat Aging. 2021;1:400–12. 33959723 10.1038/s43587-021-00051-5 PMC7610725

[47] Capasso M Diskin SJ Totaro F. Replication of GWAS-identified neuroblastoma risk loci strengthens the role of BARD1 and affirms the cumulative effect of genetic variations on disease susceptibility. Carcinogenesis. 2013;34:605–11. 23222812 10.1093/carcin/bgs380 PMC3716226

[48] Pierce BL Burgess S. Efficient design for Mendelian randomization studies: subsample and 2-sample instrumental variable estimators. Am J Epidemiol. 2013;178:1177–84. 23863760 10.1093/aje/kwt084 PMC3783091

[49] Bowden J Davey Smith G Burgess S. Mendelian randomization with invalid instruments: effect estimation and bias detection through Egger regression. Int J Epidemiol. 2015;44:512–25. 26050253 10.1093/ije/dyv080 PMC4469799

[50] Bowden J Davey Smith G Haycock PC Burgess S. Consistent estimation in mendelian randomization with some invalid instruments using a weighted median estimator. Genet Epidemiol. 2016;40:304–14. 27061298 10.1002/gepi.21965 PMC4849733

[51] Bowden J Hemani G Davey Smith G. Invited commentary: detecting individual and global horizontal pleiotropy in mendelian randomization-a job for the humble heterogeneity statistic? Am J Epidemiol. 2018;187:2681–5. 30188969 10.1093/aje/kwy185 PMC6269239

[52] Verbanck M Chen CY Neale B Do R. Detection of widespread horizontal pleiotropy in causal relationships inferred from Mendelian randomization between complex traits and diseases. Nat Genet. 2018;50:693–8. 29686387 10.1038/s41588-018-0099-7 PMC6083837

[53] Hemani G Tilling K Davey Smith G. Orienting the causal relationship between imprecisely measured traits using GWAS summary data. PLoS Genet. 2017;13:e1007081. 29149188 10.1371/journal.pgen.1007081 PMC5711033

[54] Burgess S Thompson SG. Multivariable Mendelian randomization: the use of pleiotropic genetic variants to estimate causal effects. Am J Epidemiol. 2015;181:251–60. 25632051 10.1093/aje/kwu283 PMC4325677

[55] Wang W Jiang B Sun H. Prevalence, incidence, and mortality of stroke in china: results from a nationwide population-based survey of 480 687 adults. Circulation. 2017;135:759–71. 28052979 10.1161/CIRCULATIONAHA.116.025250

[56] Guo W Li BL Zhao JY Li XM Wang LF. Causal associations between modifiable risk factors and intervertebral disc degeneration. Spine J. 2024;24:195–209. 37939919 10.1016/j.spinee.2023.10.021

[57] Powers KM Kay DM Factor SA. Combined effects of smoking, coffee, and NSAIDs on Parkinson’s disease risk. Mov Disord. 2008;23:88–95. 17987647 10.1002/mds.21782

[58] Nouri A Tetreault L Singh A Karadimas SK Fehlings MG. Degenerative cervical myelopathy: epidemiology, genetics, and pathogenesis. Spine (Phila Pa 1976). 2015;40:E675–93. 25839387 10.1097/BRS.0000000000000913

[59] Wu JC Chen Y-C Liu L. Increased risk of stroke after spinal cord injury: a nationwide 4-year follow-up cohort study. Neurology. 2012;78:1051–7. 22377807 10.1212/WNL.0b013e31824e8eaa

[60] Urban JPG Roberts S. Degeneration of the intervertebral disc. Arthritis Res Ther. 2003;5:120–30. 12723977 10.1186/ar629 PMC165040

[61] Chung KM Ho C-H Chen Y-C. Chronic pain increases the risk for major adverse cardiac and cerebrovascular events: a nationwide population-based study in Asia. Pain Med. 2020;21:1985–90. 32377670 10.1093/pm/pnaa107

[62] Lin L Lin J Qiu J. Genetic liability to multi-site chronic pain increases the risk of cardiovascular disease. Br J Anaesth. 2023;131:373–84. 37225534 10.1016/j.bja.2023.04.020

[63] Goldstein LB Bushnell CD Adams RJ. Guidelines for the primary prevention of stroke: a guideline for healthcare professionals from the American Heart Association/American Stroke Association. Stroke. 2011;42:517–84. 21127304 10.1161/STR.0b013e3181fcb238

[64] Lee CD Folsom AR Blair SN. Physical activity and stroke risk: a meta-analysis. Stroke. 2003;34:2475–81. 14500932 10.1161/01.STR.0000091843.02517.9D

[65] Hu G Sarti C Jousilahti P Silventoinen K Barengo NC Tuomilehto J. Leisure time, occupational, and commuting physical activity and the risk of stroke. Stroke. 2005;36:1994–9. 16081862 10.1161/01.STR.0000177868.89946.0c

[66] Dantas F Caires ACV Cariri GA Dantas FLR. Perioperative ischemic and hemorrhagic stroke in spine surgery: a series of 5 cases. World Neurosurg. 2021;146:E175–83. 33091642 10.1016/j.wneu.2020.10.072

[67] Yan X Pang Y Yan L. Perioperative stroke in patients undergoing spinal surgery: a retrospective cohort study. BMC Musculoskelet Disord. 2022;23:652. 35804343 10.1186/s12891-022-05591-4 PMC9264537

[68] Ishak B Abdul-Jabbar A Singla A. Intraoperative ischemic stroke in elective spine surgery: a retrospective study of incidence and risk. Spine (Phila Pa 1976). 2020;45:109–15. 31389864 10.1097/BRS.0000000000003184

[69] Park JO Lee BH Kang Y-M. Inflammatory cytokines induce fibrosis and ossification of human ligamentum flavum cells. J Spinal Disord Tech. 2013;26:E6–12. 22832553 10.1097/BSD.0b013e3182698501

[70] Lambertsen KL Biber K Finsen B. Inflammatory cytokines in experimental and human stroke. J Cereb Blood Flow Metab. 2012;32:1677–98. 22739623 10.1038/jcbfm.2012.88 PMC3434626

[71] Tueth LE Duncan RP. Musculoskeletal pain in Parkinson’s disease: a narrative review. Neurodegener Dis Manag. 2021;11:373–85. 34410146 10.2217/nmt-2021-0011 PMC8515213

[72] Baik JS Kim JY Park JH Han SW Park JH Lee MS. Scoliosis in patients with Parkinson’s disease. J Clin Neurol. 2009;5:91–4. 19587816 10.3988/jcn.2009.5.2.91 PMC2706417

[73] Chou R Qaseem A Snow V. Diagnosis and treatment of low back pain: a joint clinical practice guideline from the American College of Physicians and the American Pain Society. Ann Intern Med. 2007;147:478–91. 17909209 10.7326/0003-4819-147-7-200710020-00006

[74] Thomson S Huygen F Prangnell S. Appropriate referral and selection of patients with chronic pain for spinal cord stimulation: European consensus recommendations and e-health tool. Eur J Pain. 2020;24:1169–81. 32187774 10.1002/ejp.1562 PMC7318692

[75] Lee MS Shin BC Kong JC Ernst E. Effectiveness of acupuncture for Parkinson disease: a systematic review. Mov Disord. 2008;23:1505–15. 18618661 10.1002/mds.21993

[76] Hvingelby VS Carra RB Terkelsen MH. A pragmatic review on spinal cord stimulation therapy for Parkinson’s disease gait related disorders: gaps and controversies. Mov Disord Clin Pract. 2024;11:927–47. 38899557 10.1002/mdc3.14143 PMC11329578

[77] Pezet S McMahon SB. Neurotrophins: mediators and modulators of pain. Annu Rev Neurosci. 2006;29:507–38. 16776595 10.1146/annurev.neuro.29.051605.112929

[78] Krock E Rosenzweig DH Chabot-Doré A-J. Painful, degenerating intervertebral discs up-regulate neurite sprouting and CGRP through nociceptive factors. J Cell Mol Med. 2014;18:1213–25. 24650225 10.1111/jcmm.12268 PMC4508160

[79] Hyman C Hofer M Barde YA. BDNF is a neurotrophic factor for dopaminergic neurons of the substantia nigra. Nature. 1991;350:230–2. 2005978 10.1038/350230a0

[80] Endres M Moro MA Nolte CH Dames C Buckwalter MS Meisel A. Immune pathways in etiology, acute phase, and chronic sequelae of ischemic stroke. Circ Res. 2022;130:1167–86. 35420915 10.1161/CIRCRESAHA.121.319994

[81] Herpich F Rincon F. Management of acute ischemic stroke. Crit Care Med. 2020;48:1654–63. 32947473 10.1097/CCM.0000000000004597 PMC7540624

[82] Bloem BR Okun MS Klein C. Parkinson’s disease. Lancet. 2021;397:2284–303. 33848468 10.1016/S0140-6736(21)00218-X

[83] Tolosa E Garrido A Scholz SW Poewe W. Challenges in the diagnosis of Parkinson’s disease. Lancet Neurol. 2021;20:385–97. 33894193 10.1016/S1474-4422(21)00030-2 PMC8185633

